# Radiolabeled cyclic RGD peptides as radiotracers for tumor imaging

**DOI:** 10.1007/s41048-016-0021-8

**Published:** 2016-04-12

**Authors:** Jiyun Shi, Fan Wang, Shuang Liu

**Affiliations:** 1 Interdisciplinary Laboratory, Institute of Biophysics, Chinese Academy of Sciences, Beijing, 100101 China; 2 Medical Isotopes Research Center, Peking University, Beijing, 100191 China; 3 School of Health Sciences, Purdue University, West Lafayette, IN 47907 USA

**Keywords:** Integrin α_v_β_3_, PET and SPECT radiotracers, Tumor imaging

## Abstract

The integrin family comprises 24 transmembrane receptors, each a heterodimeric combination of one of 18α and one of 8β subunits. Their main function is to integrate the cell adhesion and interaction with the extracellular microenvironment with the intracellular signaling and cytoskeletal rearrangement through transmitting signals across the cell membrane upon ligand binding. Integrin α_v_β_3_ is a receptor for the extracellular matrix proteins containing arginine–glycine–aspartic (RGD) tripeptide sequence. The α_v_β_3_ is generally expressed in low levels on the epithelial cells and mature endothelial cells, but it is highly expressed in many solid tumors. The α_v_β_3_ levels correlate well with the potential for tumor metastasis and aggressiveness, which make it an important biological target for development of antiangiogenic drugs, and molecular imaging probes for early tumor diagnosis. Over the last decade, many radiolabeled cyclic RGD peptides have been evaluated as radiotracers for imaging tumors by SPECT or PET. Even though they are called “α_v_β_3_-targeted” radiotracers, the radiolabeled cyclic RGD peptides are also able to bind α_v_β_5_, α_5_β_1_, α_6_β_4_, α_4_β_1_, and α_v_β_6_ integrins, which may help enhance their tumor uptake due to the “increased receptor population.” This article will use the multimeric cyclic RGD peptides as examples to illustrate basic principles for development of integrin-targeted radiotracers and focus on different approaches to maximize their tumor uptake and *T*/*B* ratios. It will also discuss important assays for pre-clinical evaluations of the integrin-targeted radiotracers, and their potential applications as molecular imaging tools for noninvasive monitoring of tumor metastasis and early detection of the tumor response to antiangiogenic therapy.

## INTRODUCTION

Cancer is the second leading cause of death worldwide (Siegel et al. 2015). Most patients will survive if the cancer can be detected at the early stage. Accurate and rapid detection of rapidly growing and metastatic tumors is of great importance before they become widely spread. There are several imaging modalities available for the diagnosis of cancer, including X-ray computed tomography (CT), ultrasound (US), nuclear magnetic resonance imaging (MRI), and nuclear medicine procedures. While CT, US and MRI are better suited for anatomic analysis of solid tumors, molecular imaging with positron emission tomography (PET) and single-photon emission computed tomography (SPECT) offers significant advantages with respect to sensitivity and specificity because they are able to provide the detailed information related to biochemical changes in tumor tissues at the cellular and molecular levels (Mankoff et al. 2008; Shokeen and Anderson 2009; Tweedle 2009; Correia et al. 2011; Fani and Maecke 2012; Fani et al. 2012; Gaertner et al. 2012; Laverman et al. 2012b; Jamous et al. 2013). The most sensitive molecular imaging modalities are SPECT (~10^−10^ mol/L) and PET (10^−10^–10^−12^ mol/L) using radiotracers (Fani and Maecke 2012; Fani et al. 2012; Gaertner et al. 2012). According to their biodistribution properties, radiotracers are classified as those whose biodistribution is determined by their chemical and physical properties, and those whose ultimate distribution is determined by their receptor or enzyme binding. The latter class is called target-specific radiotracers. Peptides are often used as targeting biomolecules (BM) for receptor binding in order to achieve high tumor specificity. Many radiotracers have been developed to target the receptors overexpressed on tumor cells and/or tumor vasculature (Mankoff et al. 2008; Shokeen and Anderson 2009; Tweedle 2009; Correia et al. 2011; Fani and Maecke 2012; Fani et al. 2012; Gaertner et al. 2012; Laverman et al. 2012b; Jamous et al. 2013).

A large number of radiolabeled cyclic RGD (arginine–glycine–aspartic) peptides have been evaluated as SPECT or PET radiotracers for tumor imaging (Liu et al. 2005; Wu et al. 2005; Jia et al. 2006; Liu et al. 2006; Zhang et al. 2006; Alves et al. 2007; Dijkgraaf et al. 2007a, b; Liu et al. 2007; Wu et al. 2007; Jia et al. 2008; Li et al. 2008b; Liu et al. 2008a; Shi et al. 2008; Wang et al. 2008a, b; Liu et al. 2009a, b; Shi et al. 2009a, b, c; Chakraborty et al. 2010; Kubas et al. 2010; Dumont et al. 2011; Jia et al. 2011; Shi et al. 2011a, b; Zhou et al. 2011b; Nwe et al. 2012; Pohle et al. 2012; Zhou et al. 2012; Ji et al. 2013a, b; Li et al. 2013; Simecek et al. 2013; Tsiapa et al. 2013; Maschauer et al. 2014; Yang et al. 2014; Zheng et al. 2015). Many excellent review articles have appeared to cover their nuclear medicine applications (D’Andrea et al. 2006; Liu 2006; Meyer et al. 2006; Beer and Schwaiger 2008; Cai and Chen 2008; Liu et al. 2008b; Liu 2009; Stollman et al. 2009; Beer and Chen 2010; Chakraborty and Liu 2010; Dijkgraaf and Boerman 2010; Haubner et al. 2010; Beer et al. 2011; Michalski and Chen 2011; Zhou et al. 2011a; Danhier et al. 2012; Tateishi et al. 2012. This article is not intended to be an exhaustive review of current literature on radiolabeled cyclic RGD peptides. Instead, it will use the multimeric cyclic RGD peptides to illustrate some basic principles for new radiotracer development and to address some important issues associated with integrin-targeted radiotracers. It will focus on different approaches to maximize the tumor uptake and *T*/*B* ratios. Authors would apologize to those whose work has not been cited in this article.

## RADIOTRACER DESIGN

### Integrin-targeted radiotracer

**Fig. 1:**
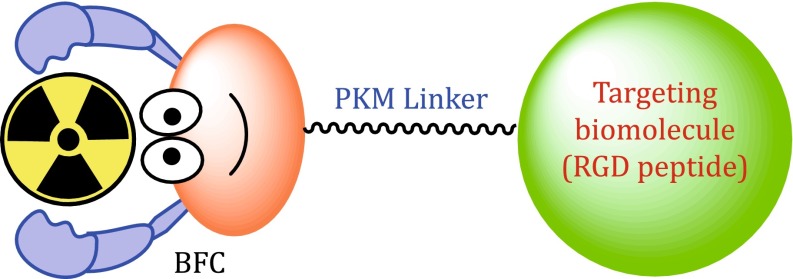
Schematic presentation of the integrin α_v_β_3_-targeted radiotracers. The cyclic RGD peptide is used as the targeting biomolecule (BM) to carry the isotope into the tumor tissue. BFC is used to attach the isotope to the targeting biomolecule. PKM linker is utilized to modify its pharmacokinetics

Figure 1 shows the schematic construction of an integrin-targeted radiotracer (Liu 2006, 2009). The cyclic RGD peptide serves as a “vehicle” to carry the isotope to integrins expressed on both tumor cells and activated endothelial cells of tumor neovasculature. BFC is a bifunctional coupling agent to attach the appropriate radionuclide to a cyclic RGD peptide (Liu and Edwards 2001; Liu 2004, 2008; Liu and Chakraborty 2011). The pharmacokinetic modifying (PKM) linker is often used to improve excretion kinetics of radiotracers (Liu and Edwards 2001; Liu 2004, 2008). For a new radiotracer to be successful in clinics, it must show clinical indications for several of high-incidence tumor types (namely breast, lung, and prostate cancers). Renal excretion is necessary in order to maximize the tumor-to-background (*T*/*B*) ratios. The main objective of tumor imaging is to achieve the following goals: (1) to detect the presence of tumor at early stage, (2) to distinguish between benign and malignant tumors, (3) to follow the tumor growth and tumor response to a specific therapy (chemotherapy, radiation therapy, or combination thereof), (4) to predict success or failure of a specific therapeutic regimen, and (5) to access the prognosis of a particular tumor.

### Radionuclide

The choice of radionuclide depends largely on the modality for tumor imaging. More than 80% of radiotracers for SPECT in nuclear medicine departments are ^99m^Tc compounds due to optimal nuclear properties of ^99m^Tc and its easy availability at low cost (Liu and Edwards 2001; Liu 2004, 2008; Liu and Chakraborty 2011). The 6-h half-life is long enough to allow radiopharmacists to carry out radiosynthesis and for physicians to collect clinically useful images. At the same time, it is short enough to permit administration of 20–30 mCi of ^99m^Tc without imposing a significant radiation dose to the patients. ^18^F is a cyclotron-produced isotope suitable for PET. It has a half-life of 110 min. Despite its short half-life, the availability of preparative modules makes ^18^F radiotracers more accessible to clinicians (Anderson et al. 2003). ^64^Cu is another PET isotope to develop target-specific radiotracers. It has a half-life of 12.7 h and a β^+^ emission (18%, *E*_max_ = 0.655 MeV). Despite poor nuclear properties, ^64^Cu is a viable alternative to ^18^F for research programs that wish to incorporate high sensitivity and spatial resolution of PET, but cannot afford to maintain the expensive isotope production infrastructure (Anderson et al. 2003). ^68^Ga is generator-produced PET isotope with the half-life of 68 min. The ^68^Ge–^68^Ga generator can be used for more than a year. ^68^Ga could become as useful for PET as ^99m^Tc for SPECT (Maecke et al. 2005). The ^68^Ga-labeled somatostatin analogs have been studied for PET imaging of somatostatin-positive tumors in both pre-clinical animal models and cancer patients (Henze et al. 2005; Koukouraki et al. 2006a, b). Gallium chemistry and related nuclear medicine applications have been reviewed recently (Maecke et al. 2005).

### Bifunctional coupling agent (BFC)

**Fig. 2:**
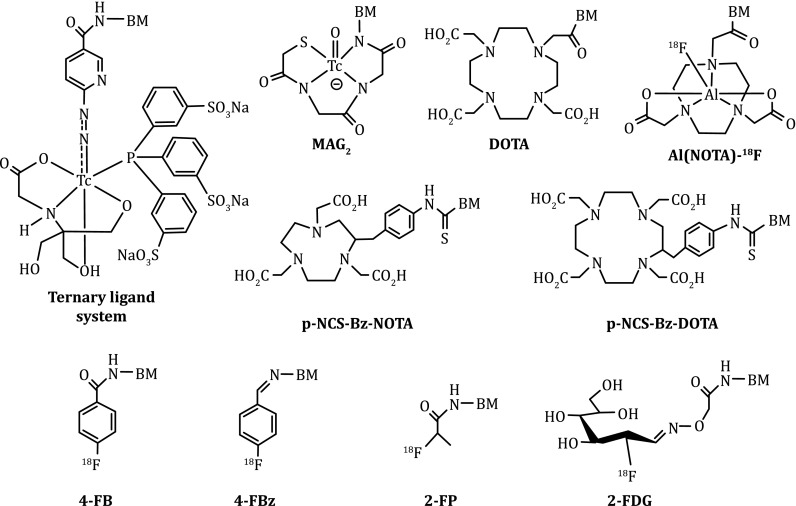
Examples of BFCs useful for radiolabeling of cyclic RGD peptides. HYNIC and MAG_2_ are useful for ^99m^Tc-labeling while DOTA, NOTA, and their derivatives are better suited for chelation of ^64^Cu and ^68^Ga. For ^18^F-labeling, 4-FB, 4-FBz, 2-FP, and 2-FDG are often used as prosthetic groups. The Al(NOTA) chelate is highly efficient for radiosynthesis of ^18^F radiotracers using a kit formulation

The choice of BFC depends on the radionuclide (Liu 2004, 2008; Liu and Chakraborty 2011). Among various BFCs for ^99m^Tc-labeling, 6-hydazinonicotinic acid (Fig. 2: HYNIC) is of great interest due to its high efficiency (rapid radiolabeling and high radiolabeling yield), the high solution stability of its ^99m^Tc complexes, and the easy use of co-ligands for modification of biodistribution properties of ^99m^Tc radiotracers (Liu 2004, 2005, 2008; Liu and Chakraborty 2011). In contrast, DOTA (1,4,7,10-tetraazacyclododecane-1,4,7,10-tetraacetic acid), NOTA (1,4,7-triazacyclononane-1,4,7-triacetic acid), and their derivatives (Fig. 2) have been widely used for ^68^Ga/^64^Cu-labeling of biomolecules due to the high hydrophilicity and in vivo stability of its ^68^Ga/^64^Cu chelates (Anderson et al. 2003; Maecke et al. 2005; Shokeen and Anderson 2009). Organic prosthetic groups (Fig. 2: 4-FB, 4-FBz, 2-FP, and 2-FDG) are often needed for ^18^F-labeling (Dolle 2005; Li et al. 2007, 2008a; Glaser et al. 2008; Hausner et al. 2008; Hohne et al. 2008; Mu et al. 2008; Becaud et al. 2009; Namavari et al. 2009; Vaidyanathan et al. 2009; Jacobson and Chen 2010; Liu et al. 2010; Wangler et al. 2010; Jacobson et al. 2011; Schirrmacher et al. 2013). However, recent results indicate that the Al(NOTA) chelates is more efficient for routine radiosynthesis of ^18^F radiotracers using the kit formulation (McBride et al. 2009, 2010, 2012; D’Souza et al. 2011; Lang et al. 2011; Liu et al. 2011; Laverman et al. 2010, 2012a).

### Integrins as molecular targets for tumor imaging

Angiogenesis is a requirement for tumor growth and metastasis (Hwang and Varner 2004; Weigelt et al. 2005). The angiogenic process depends on the vascular endothelial cell migration and invasion, and is regulated by cell adhesion receptors. Integrins are such a family of receptors that facilitate the cellular adhesion to and the migration on extracellular matrix proteins, and regulate the cellular entry and withdraw from the cell cycle (Albelda et al. 1990; Falcioni et al. 1994; Carreiras et al. 1996; Bello et al. 2001; Sengupta et al. 2001; Cooper et al. 2002; Zitzmann et al. 2002; Hwang and Varner 2004; Jin and Varner 2004; Weigelt et al. 2005; Sloan et al. 2006; Zhao et al. 2007; Hodivala-Dilke 2008; Barczyk et al. 2010; Taherian et al. 2011; Gupta et al. 2012; Sheldrake and Patterson 2014). The integrin family comprises 24 transmembrane receptors (Table 1) (Sheldrake and Patterson 2014). Their main function is to integrate the cell adhesion and interaction with the extracellular microenvironment with the intracellular signaling and cytoskeletal rearrangement through transmitting signals across the cell membrane on ligand binding. Many integrins are crucial to the tumor initiation, progression, and metastasis. Among the 24 members, the α_v_β_3_ is studied most extensively for its role in tumor angiogenesis and metastasis (Albelda et al. 1990; Falcioni et al. 1994; Carreiras et al. 1996; Bello et al. 2001; Sengupta et al. 2001; Cooper et al. 2002; Zitzmann et al. 2002; Hwang and Varner 2004; Jin and Varner 2004; Weigelt et al. 2005; Sloan et al. 2006; Zhao et al. 2007; Hodivala-Dilke 2008; Barczyk et al. 2010; Taherian et al. 2011; Gupta et al. 2012). It is not surprising that radiolabeled cyclic RGD peptides are often called “α_v_β_3_–targeted” radiotracers in majority of the literature (D’Andrea et al. 2006; Liu 2006; Meyer et al. 2006; Beer and Schwaiger 2008; Cai and Chen 2008; Liu et al. 2008b; Liu 2009; Stollman et al. 2009; Beer and Chen 2010; Chakraborty and Liu 2010; Dijkgraaf and Boerman 2010; Haubner et al. 2010; Beer et al. 2011; Michalski and Chen 2011; Zhou et al. 2011a; Danhier et al. 2012; Tateishi et al. 2012).

**Table 1:** Natural integrin ligands and their corresponding recognition peptide sequences

Integrins	Recognition sequence	Natural ligands
α_v_β_1_, α_v_β_3_, α_v_β_5_, α_v_β_6_, α_v_β_8_, α_5_β_1_, α_8_β_1_, α_IIb_β_3_	RGD	Vitronectin, fibronectin, osteopontin, fibrinogen
α_4_β_1_, α_9_β_1_, α_4_β_7_, α_E_β_7_, α_L_β_2_, α_M_β_2_, α_X_β_2_, α_D_β_2_	LDV and related sequences	Fibronectin, vascular cell adhesion molecule 1, mucosal addressin cell adhesion molecule 1, intercellular cell adhesion molecule 1
α_1_β_1_, α_2_β_1_, α_10_β_1_, α_11_β_1_	GFOGER	Collagen, laminin
α_3_β_1_, α_6_β_1_, α_7_β_1_, α_6_β_4_	Other	Laminin

Table were adapted from Sheldrake and Patterson (2014)

The changes in the α_v_β_3_ expression levels and activation state have been well documented during tumor growth and metastasis (Hwang and Varner 2004; Weigelt et al. 2005; Sloan et al. 2006; Zhao et al. 2007; Hodivala-Dilke 2008; Barczyk et al. 2010; Gupta et al. 2012). The α_v_β_3_ is expressed in low levels on epithelial cells and mature endothelial cells, but it is highly expressed in many solid tumors, which include osteosarcomas, glioblastoma, melanomas, and carcinomas of lung and breast (Albelda et al. 1990; Falcioni et al. 1994; Carreiras et al. 1996; Bello et al. 2001; Sengupta et al. 2001; Cooper et al. 2002; Zitzmann et al. 2002; Hwang and Varner 2004; Jin and Varner 2004; Weigelt et al. 2005; Sloan et al. 2006; Zhao et al. 2007; Hodivala-Dilke 2008; Barczyk et al. 2010; Taherian et al. 2011; Gupta et al. 2012). Studies show that α_v_β_3_ is overexpressed on tumor cells and activated endothelial cells of tumor neovasculature (Pilch et al. 2002; Taherian et al. 2011). It is believed that the α_v_β_3_ expressed on endothelial cells modulate cell adhesion and migration during angiogenesis, while the α_v_β_3_ overexpressed on carcinoma cells potentiate metastasis by facilitating invasion and movement of tumor cells across blood vessels (Sloan and Anderson 2002; Minn et al. 2005; Dittmar et al. 2008; Lorger et al. 2009; Omar et al. 2010). It has been shown that the α_v_β_3_ expression levels correlate with the potential for metastasis and aggressiveness of tumors, including glioma, melanoma, and carcinomas of the breast and lungs (Zhao et al. 2007; Hodivala-Dilke 2008). The α_v_β_3_ is considered as an important biological target to develop antiangiogenic drugs (Gottschalk and Kessler 2002; Kumar 2003; Jin and Varner 2004; D’Andrea et al. 2006) and molecular imaging probes for diagnosis of tumors (D’Andrea et al. 2006; Meyer et al. 2006; Liu 2006, 2009; Beer and Schwaiger 2008; Cai and Chen 2008; Liu et al. 2008b; Stollman et al. 2009; Beer and Chen 2010; Chakraborty and Liu 2010; Dijkgraaf and Boerman 2010; Haubner et al. 2010; Beer et al. 2011; Michalski and Chen 2011; Zhou et al. 2011a; Danhier et al. 2012; Tateishi et al. 2012).

### Cyclic RGD peptides as targeting biomolecules

**Fig. 3:**
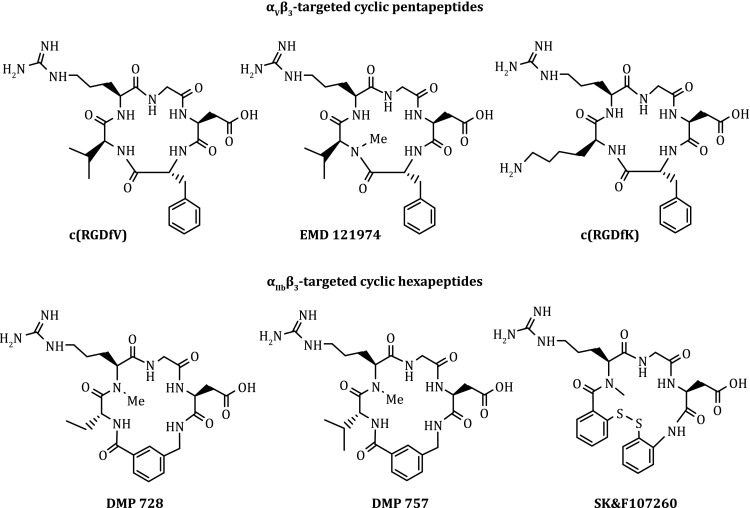
Examples of monomeric cyclic RGD peptides as targeting biomolecules for the development of α_v_β_3_-targeted radiotracers. EMD121974 has been under clinical investigations as an “orphan drug” for treatment of glioblastoma either stand-alone or in combination with radiation therapy. DMP728 and DMP757 were originally developed as anti-thrombotic agents

The α_v_β_3_ is a receptor for the extracellular matrix proteins with the exposed RGD tripeptide sequence. Theoretically, both linear and cyclic RGD peptides can be used as targeting biomolecules. A major drawback of linear RGD peptides are their low binding affinity (IC_50_ > 100 nmol/L), lack of specificity (α_v_β_3_ vs. α_IIB_β_3_), and rapid degradation by proteases in serum. Cyclization of RGD peptides via the linkers, such as S-S disulfide, thioether, and rigid aromatic rings, leads to the increased receptor binding affinity and selectivity (Aumailley et al. 1991; Gurrath et al. 1992; Müller et al. 1992; Haubner et al. 1996). It seems that the α_IIB_β_3_ is less sensitive to variations in the RGD peptide backbone and can accommodate a larger distance or spacer than α_IIB_β_3_ and α_v_β_5_ (Pfaff et al. 1994). Incorporation of the RGD sequence into a cyclic pentapeptide framework (Fig. 3: c(RGDfV) and EMD121974) could significantly increase the binding affinity and selectivity of α_v_β_3_/α_v_β_5_ over α_IIb_β_3_ (Aumailley et al. 1991; Gurrath et al. 1992; Müller et al. 1992; Pfaff et al. 1994; Haubner et al. 1996). The addition of a rigid aromatic ring into the cyclic hexapeptide structure (Fig. 3: DMP728 and DMP757) enhances the binding affinity of α_IIB_β_3_ (Liu et al. 2010; Jacobson et al. 2011; Danhier et al. 2012). The structure–activity studies indicated that the amino acid residue in position 5 has little impact on α_v_β_3_/α_v_β_5_ binding affinity (Aumailley et al. 1991; Gurrath et al. 1992; Müller et al. 1992; Haubner et al. 1996). The valine (V) residue in c(RGDfV) can be replaced by lysine (K) or glutamic acid (E) to afford c(RGDfK) and c(RGDfE), respectively, without changing their α_v_β_3_/α_v_β_5_ binding affinity.

**Fig. 4:**
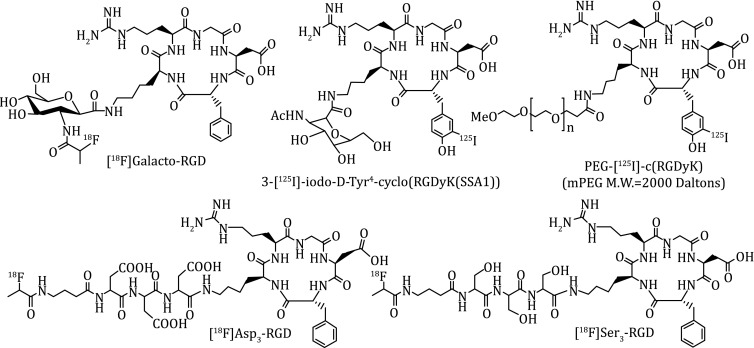
Examples of the radiolabeled monomeric cyclic RGD peptides as radiotracers

Figure 4 shows several examples of monomeric cyclic RGD peptides that have high affinity for α_v_β_3_ and α_v_β_5_. Among the radiotracers evaluated in pre-clinical tumor-bearing models, [^18^F]Galacto-RGD (Fig. 4: 2-[^18^F]fluoropropanamide c(RGDfK(SAA); SAA = 7-amino-l-glyero-l-galacto-2,6-anhydro-7-deoxyheptanamide) was the first one under clinical investigation for visualization of α_v_β_3_ expression in cancer patients (Beer et al. 2005; 2007, 2008; Haubner et al. 2005). The results from imaging studies in cancer patients showed that there was sufficient α_v_β_3_ for PET imaging. The tumor uptake of [^18^F]Galacto-RGD correlates with the α_v_β_3_ levels in cancer patients (Haubner et al. 2005; Beer et al. 2007, 2008). However, the radiotracers derived from monomeric cyclic RGD peptides all had low tumor uptake with *T*/*B* ratios because of their relatively low α_v_β_3_ binding affinity.

It must be noted that cyclic RGD peptides bind not only α_v_β_3_ but also other integrins. While the α_v_β_3_ plays pivotal role in the tumor growth and progression, α_IIB_β_3_ is critical for the platelet aggregation during thrombosis formation. The interaction between α_v_β_3_ and α_IIb_β_3_ facilitates the adhesion of tumor cells to the vasculature and often leads to metastasis (Felding-Habermann et al. 1996; Bakewell et al. 2003). The α_v_β_5_ is very similar to α_v_β_3_ in the ligand binding site region and has a similar expression pattern and function to those of α_v_β_3_. Both α_v_β_5_ and α_v_β_3_ are highly expressed on the activated endothelial cells and have similar roles in angiogenesis, promoting angiogenic response to different growth factors (Bakewell et al. 2003; Goodman et al. 2012). The α_v_β_5_ has been shown to overexpress on a wide range of tumor types (Goodman et al. 2012; Boger et al. 2014). A number of tumors co-express α_v_β_3_ and α_v_β_5_ (Sung et al. 1998; Erdreich-Epstein et al. 2000; Graf et al. 2003; Humphries et al. 2006; Monferran et al. 2008; Bianchi-Smiraglia et al. 2013; Roth et al. 2013; Vogetseder et al. 2013; Boger et al. 2014; Navarro-Gonzalez et al. 2015), because both engage the same ECM ligands and activate complementary cell signaling pathways in order to promote tumor progression (Sung et al. 1998; Bianchi-Smiraglia et al. 2013). It was also reported that the tumor cell expression of α_v_β_3_, α_v_β_5_, α_5_β_1_, α_6_β_4_, α_4_β_1_, and α_v_β_6_ is correlated with the progression of various tumors (Vogetseder et al. 2013; Boger et al. 2014). The structures of other RGD-binding integrins (α_v_β_6_, α_v_β_8_, α_v_β_1_ and α_8_β_1_) have not yet been studied in details (Sheldrake and Patterson 2014).

## MAXIMIZING BINDING AFFINITY VIA MULTIMERIZATION

**Fig. 5:**
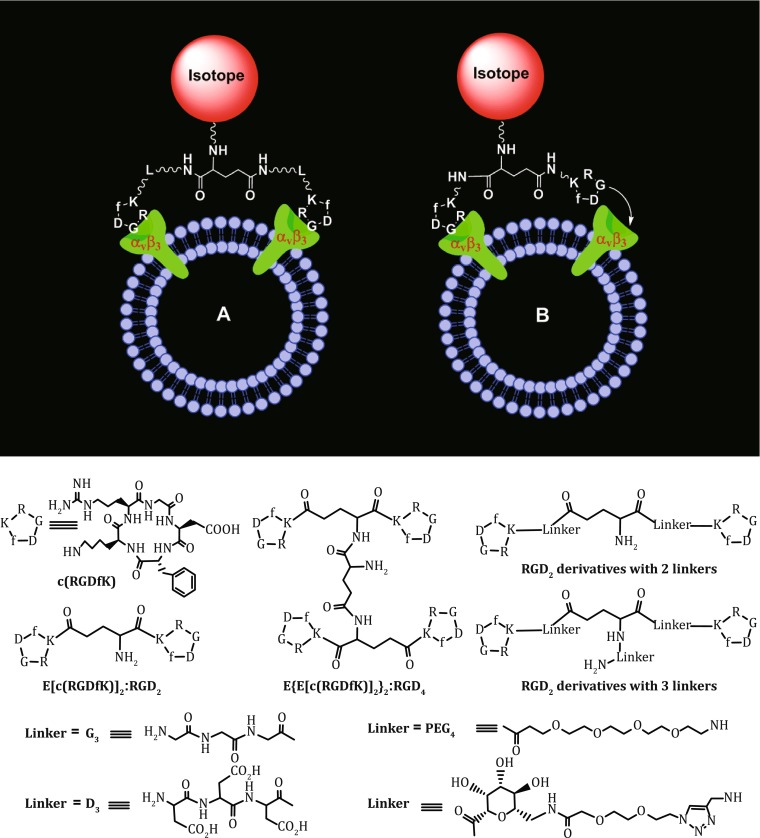
**Top**: Schematic illustration of the interactions between cyclic RGD peptide dimers and α_v_β_3_. **A** The distance between two RGD motifs is not long enough for simultaneous integrin α_v_β_3_ binding. However, the RGD concentration is “locally enriched” in the vicinity of neighboring integrin α_v_β_3_ once the first RGD motif is bound. **B** The distance between two RGD motifs is long due to the presence of two linkers (L). As a result, the cyclic RGD dimer is able to bind integrin α_v_β_3_ in a “bivalent” fashion. In both cases, the end-result would be higher integrin α_v_β_3_ binding affinity for the multimeric cyclic RGD peptides. **Bottom**: Selected cyclic RGD peptide dimers and tetramers useful for development of α_v_β_3_-targeted radiotracers. The D_3_, G_3_, PEG_4_, and sugar linkers are used to increase the distance between two RGD motifs and to improve radiotracer excretion kinetics from non-cancerous organs

**Fig. 6:**
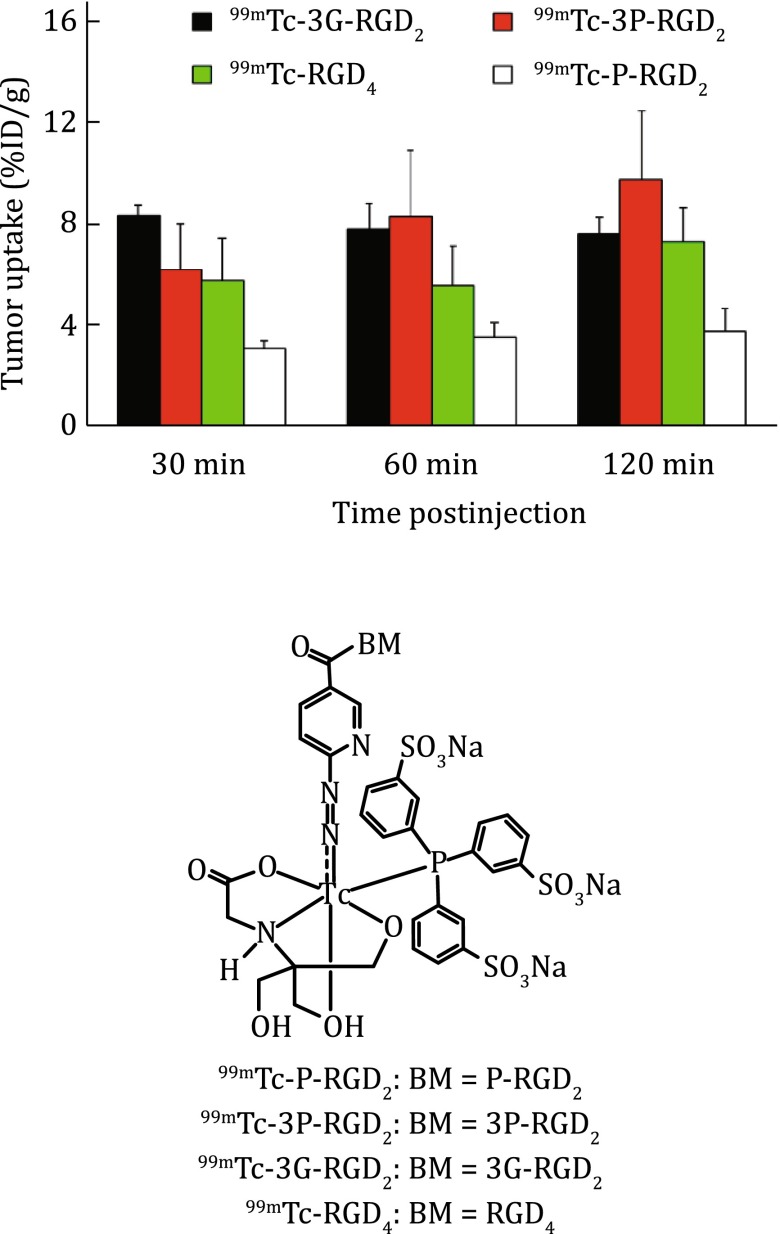
Direct comparison of tumor uptake for ^99m^Tc-P-RGD_2_, ^99m^Tc-3G-RGD_2_, ^99m^Tc-3P-RGD_2_, and ^99m^Tc-RGD_4_ in athymic nude mice bearing MDA-MB-435 breast cancer xenografts. The biodistribution data were adapted from Shi et al. (2008) and Wang et al. (2008b)

The multivalent concept has been used to develop radiotracers with the increased tumor-targeting capability. For example, E[c(RGDfK)]_2_ (RGD_2_) was the first cyclic RGD dimer for development of diagnostic (^99m^Tc) and therapeutic (^90^Y and ^64^Cu) radiotracers (Liu et al. 2001a; b; 2005, 2006, 2007, 2008a, 2015; Jia et al. 2006, 2008). RGD tetramers RGD_4_ was also used to develop SPECT and PET radiotracers (Wu et al. 2005; Liu et al. 2007, 2008a). Both the in vitro assays and biodistribution data showed that the radiolabeled (^99m^Tc, ^18^F, and ^64^Cu) multimeric cyclic RGD peptides have higher α_v_β_3_ binding affinity and better tumor uptake than their monomeric analogs (Liu et al. 2008b; Liu 2009). It is important to note that multimeric RGD peptides are not necessarily multivalent (Liu et al. 2008b; Chakraborty et al. 2010). Two factors (Fig. 5: bivalency and enhanced local RGD concentration) contribute to the high α_v_β_3_ binding affinity of cyclic RGD peptides (Liu et al. 2008b; Chakraborty et al. 2010). The concentration factor exists in all multimeric RGD peptides regardless of the linker length. Given the short distance (6 bonds excluding side-arms of K-residues) between two RGD motifs in E[c(RGDfK)]_2_ and E[c(RGDyK)]_2_, it is unlikely that they would bind to two adjacent α_v_β_3_ sites simultaneously. However, the binding of one RGD motif to α_v_β_3_ will increase the “local concentration” of second RGD motif in the vicinity of α_v_β_3_ sites (Fig. 5B). The concentration factor may explain the higher tumor uptake of radiolabeled (^99m^Tc, ^111^In, ^90^Y, ^18^F, and ^64^Cu) E[c(RGDfK)]_2_ and E[c(RGDyK)]_2_ than their monomeric derivatives (Beer and Chen 2010; Chakraborty and Liu 2010; Dijkgraaf and Boerman 2010; Beer et al. 2011; Michalski and Chen 2011; Zhou et al. 2011a). The key for bivalency is the distance between two cyclic RGD motifs. For example, this distance is 38 bonds in PEG_4_-E[c(RGDfK(PEG_4_))]_2_ (3P-RGD_2_: PEG_4_ = 15-amino-4,7,10,13-tetraoxapentadecanoic acid), and 26 bonds G_3_-E[c(RGDfK(G_3_))]_2_ (3G-RGD_2_: G_3_ = Gly-Gly-Gly), which are long enough for them to achieve the bivalency. As a result, HYNIC-3P-RGD_2_ (IC_50_ = 60 ± 3 nmol/L) and HYNIC-3G-RGD_2_ (IC_50_ = 59 ± 3 nmol/L) have much higher α_v_β_3_ binding affinity than HYNIC-P-RGD_2_ (P-RGD_2_ = PEG_4_-E[c(RGDfK)]_2_: (IC_50_ = 89 ± 7 nmol/L)) (Shi et al. 2008; Wang et al. 2008b). ^99m^Tc-3P-RGD_2_ and ^99m^Tc-3G-RGD_2_ had higher breast tumor uptake than ^99m^Tc-P-RGD_2_ (Fig. 6) (Shi et al. 2008; Wang et al. 2008b). Since the tumor uptake of ^99m^Tc-3P-RGD_2_ and ^99m^Tc-3P-RGD_2_ is comparable to that of ^99m^Tc-RGD_4_ suggests that the contribution from “concentration factor” may not be as significant as that from the “bivalency.”

## MAXIMIZING RADIOTRACER UPTAKE BY TARGETING MULTIPLE RECEPTORS

**Fig. 7:**
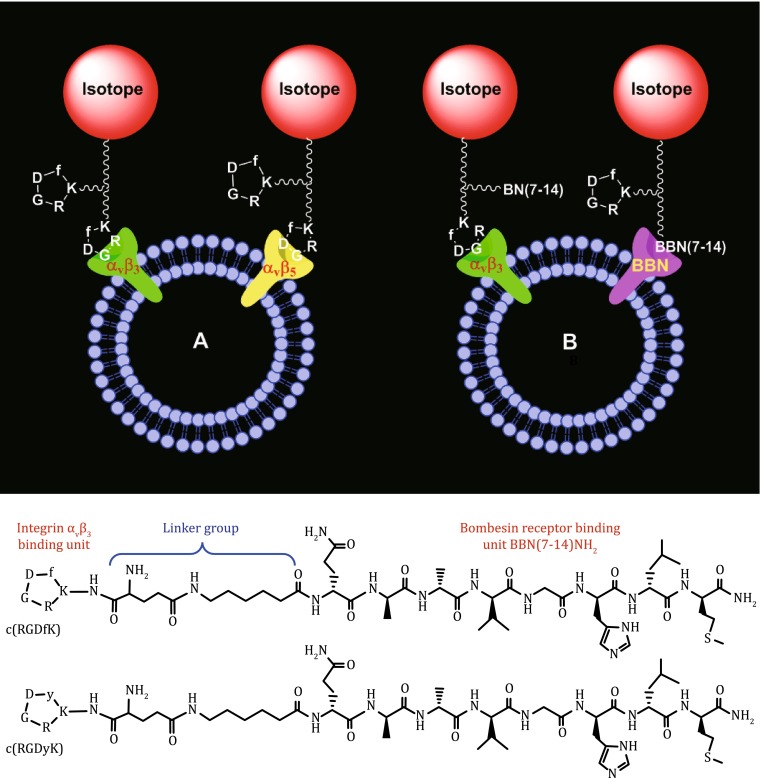
**Top**: Schematic presentation of the interactions between the dimeric cyclic RGD peptide to target two or more integrins (such as α_v_β_3_, α_v_β_5_, α_5_β_1_, α_6_β_4_, α_4_β_1_, and α_v_β_6_). **B** Schematic illustration of the interactions between the bifunctional peptide and two different receptors (α_v_β_3_ and BBN receptor). By targeting two different receptors, the radiotracer will have more opportunities to localize in the tumor because of the increased receptor population. The two targeted receptors (e.g., α_v_β_3_/α_v_β_5_ or α_v_β_3_/BBN) must be co-localized and the distance between them must be short for the bifunctional radiotracer to achieve “simultaneous receptor binding.” **Bottom**: Selected examples of bifunctional peptides containing c(RGDfK)/c(RGDyK) and Aca-BBN(7–14)NH_2_ (ε-aminocaproic acid-Gln-Trp-Ala-Val-Gly-His-Leu-Met-NH_2_)

Two most important factors affecting the radiotracer tumor uptake are receptor binding affinity and receptor population. The receptor binding affinity is critically important for selective tumor localization and tumor uptake of radiolabeled cyclic RGD peptides (Liu et al. 2008b). The receptor population is equally important for the receptor-based molecular imaging. It will not be possible to image the tumor if that it has very limited or no receptor expression even if the receptor ligand has high receptor binding affinity. There are two approaches to maximize the target population. The first approach (Fig. 7A) involves the use of the same cyclic RGD peptide to target two or more integrins (such as α_v_β_3_, α_v_β_5_, α_5_β_1_, α_6_β_4_, α_4_β_1_, and α_v_β_6_). Another approach (Fig. 7B) involves the use of a bifunctional peptide that is able to target two different receptors, such as α_v_β_3_ and bombesin (BBN) receptor. By targeting two different receptors, the radiotracer will have more opportunities to localize in the tumor due to the larger populations of two receptors than that of a single receptor. The so-called “bivalent heterodimers” (Fig. 7) has been used to target the α_v_β_3_ and BBN receptors (Li et al. 2008c; Liu et al. 2009c, d). The xenografted PC-3 and MDA-MB-435 tumor-bearing models were used to evaluate their tumor-targeting capability and biodistribution properties. It is well-established that the xenografted PC-3 tumors have low α_v_β_3_ expression (Zhou et al. 2011b; Ji et al. 2013c). It was also shown that the xenografted MDA-MB-435 tumor has little BBN receptor expression (Liu et al. 2009c, d). Therefore, both PC-3 and MDA-MB-435 tumor-bearing models are not appropriate to prove the concept of “bivalent heterodimers.” For the bifunctional radiotracers to achieve the bivalency, the α_v_β_3_ and BBN receptors must be co-localized and the distance between them must be short. Otherwise, it would not be advantageous even if they might be able to target both individual receptors. Unfortunately, there is lack of concrete experimental data to demonstrate if the c(RGDfK)-BBN(7–14) and c(RGDyK)-BBN(7–14) conjugates are “bivalent” for tumor targeting, and whether there is indeed a “synergetic effect” between the cyclic RGD and BBN(7–14) peptides. Another challenge associated with the “bifunctional heterodimer concept” is which binding unit actually contributes to the radiotracer tumor uptake.

## INTEGRIN AND RGD SPECIFICITY

### Integrin specificity

**Fig. 8:**
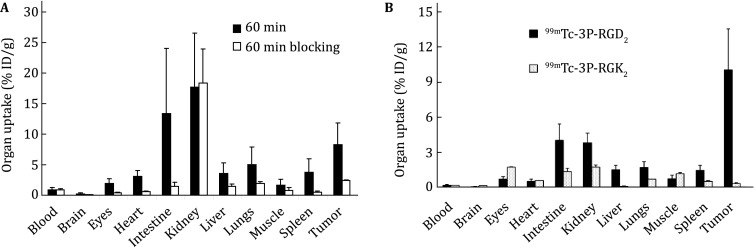
**A** Comparison of organ uptake (%ID/g) for ^99m^Tc-2P-RGD_2_ in athymic nude mice bearing U87MG glioma xenografts in the absence or presence of excess RGD_2_ at 60 min p.i. Co-injection of excess RGD_2_ resulted in significant reduction in the uptake of ^99m^Tc-2P-RGD_2_ in the tumor and normal organs. **B** Comparison of the 60-min biodistribution data of ^111^In-3P-RGD_2_ and ^111^In-3P-RGK_2_ in athymic nude mice bearing U87MG glioma xenografts. The low tumor uptake for ^111^In-3P-RGK_2_ indicates that the radiolabeled cyclic RGD dimers are RGD-specific. The experimental data were adapted from Shi et al. (2011a)

Blocking experiment (Fig. 8A) has been used to demonstrate the α_v_β_3_ specificity of radiolabeled RGD peptides with a known α_v_β_3_ antagonist (e.g., c(RGDfK) or RGD_2_) as the blocking agent. This experiment is often performed by biodistribution or imaging (PET or SPECT). The blocking agent is pre- or co-injected with the radiotracer. Co-injection or pre-injection of excess blocking agents (such as RGD_2_) will result in partial or complete blockage of the radiotracer tumor uptake (Fig. 8B). It is important to note that there is also a significant reduction in radiotracer uptake in the α_v_β_3_-positive organs (e.g., eyes, intestine, kidneys, lungs, liver, muscle, and spleen). The normal organ uptake is consistent with the β_3_ and CD31 staining data for the liver, kidneys, and lungs from the tumor-bearing athymic nude mice.

### RGD specificity

There are several ways to determine the RGD specificity of radiolabeled cyclic RGD peptides, including: (1) the in vitro binding assay using ^125^I-echistatin as the integrin-specific radioligand (Zhang et al. 2006; Wu et al. 2007; Wang et al. 2008b; Shi et al. 2009c), (2) the in vitro tissue or cellular immunohistochemical (IHC) staining assay using fluorescent probes (Zheng et al. 2014), (3) the in vivo imaging experiment (PET or SPECT) (Zhang et al. 2006; Wu et al. 2007; Wang et al. 2008b; Shi et al. 2009c), and (4) the biodistribution study (Shi et al. 2009a, 2011a, b; Chakraborty et al. 2010). In all cases, a nonsense peptide with the “scrambled sequence” will be used to prepare the corresponding radiotracer or fluorescent probe. For example, 3P-RGK_2_ is the nonsense peptide with the composition identical to that of 3P-RGD_2_. The α_v_β_3_ binding affinity of DOTA-3P-RGK_2_ (IC_50_ = 596 ± 48 nmol/L) was >20× lower than that of DOTA-3P-RGD_2_ (IC_50_ = 29 ± 4 nmol/L). Similar results were also seen with FITC-3P-RGK_2_ (IC_50_ = 589 ± 73 nmol/L) and FITC-3P-RGD_2_ (IC_50_ = 32 ± 7 nmol/L). Because of the low α_v_β_3_ affinity of DOTA-3P-RGK_2_ (Chakraborty et al. 2010; Shi et al. 2011a, b), ^111^In(DOTA-3P-RGK_2_) had significantly lower (*p* < 0.01) uptake than ^111^In(DOTA-3P-RGD_2_) in the xenografted breast tumors and the α_v_β_3_-positive normal organs, such as eyes, intestine, liver, lungs, and spleen (Fig. 8B) (Shi et al. 2011a). These results clearly show that the uptake of radiolabeled cyclic RGD peptides in tumors and some normal organs is indeed α_v_β_3_-specific.

## LINEAR RELATIONSHIP BETWEEN RADIOTRACER TUMOR UPTAKE AND Α_V_Β_3_ EXPRESSION

It has been shown that the radiolabeled cyclic RGD peptides are useful for non-invasive imaging of tumors in cancer patients (Beer et al. 2005, 2007, 2008; Haubner et al. 2005). It is the total α_v_β_3_ level that will contribute the tumor uptake of a α_v_β_3_-targeted radiotracer. The capability to visualize the α_v_β_3_ expression provides new opportunities to characterize the tumor angiogenesis noninvasively, to select appropriate patients for antiangiogenic treatment, and to monitor the tumor response to antiangiogenic drugs. However, there were only a few reports on the correlation between the α_v_β_3_ expression levels and radiotracer tumor uptake (Beer et al. 2005, 2007, 2008; Haubner et al. 2005; Zhang et al. 2006).

**Fig. 9:**
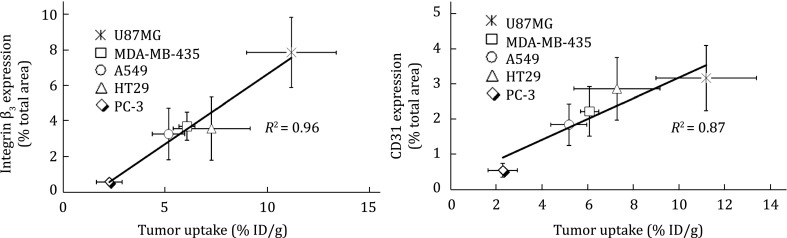
Relationship between the tumor uptake (%ID/g: radioactivity density) and relative β_3_ or CD31 levels in five xenografted tumors (U87MG, MDA-MB-435, A549, HT29, and PC-3). The total β_3_ expression was represented by the percentage of red area over total area in each slice of tumor tissue. Each data point was derived from at least 15 different areas of same tissue (×100 magnification). Experiments were repeated three times independently with similar results. The experimental data were adapted from Zhou et al. (2011b)

^99m^Tc-3P-RGD_2_ was studied for its capability to monitor the α_v_β_3_ expression in five different tumor-bearing animal models (U87MG, MDA-MB-435, A549, HT29, and PC-3). IHC staining was performed to determine the α_v_β_3_ and CD31 (a biomarker for tumor vasculature) expression levels in xenografted U87MG, MDA-MB-435, A549, HT29, and PC-3 tumor tissues (Zhou et al. 2011b). It was found that the total α_v_β_3_ expression levels on the tumor cells and tumor neovasculature follow the general ranking trend: U87MG > MDA-MB-435 = A549 = HT29 > PC-3. In contrast, the CD31 expression levels follow the general ranking order of U87MG = HT29 > MDA-MB-435 = A549 > PC-3 (Fig. 9). More importantly, there is an excellent relationship between the tumor uptake and the α_v_β_3_ expression levels (Zhou et al. 2011b). The linear relationship between the tumor uptake (%ID/g) and α_v_β_3_ density suggests that ^99m^Tc-3P-RGD_2_ is useful for non-invasive monitoring of the α_v_β_3_ expression levels in cancer patients.

## MONITORING TUMOR RESPONSE TO ANTIANGIOGENIC THERAPY

**Fig. 10:**
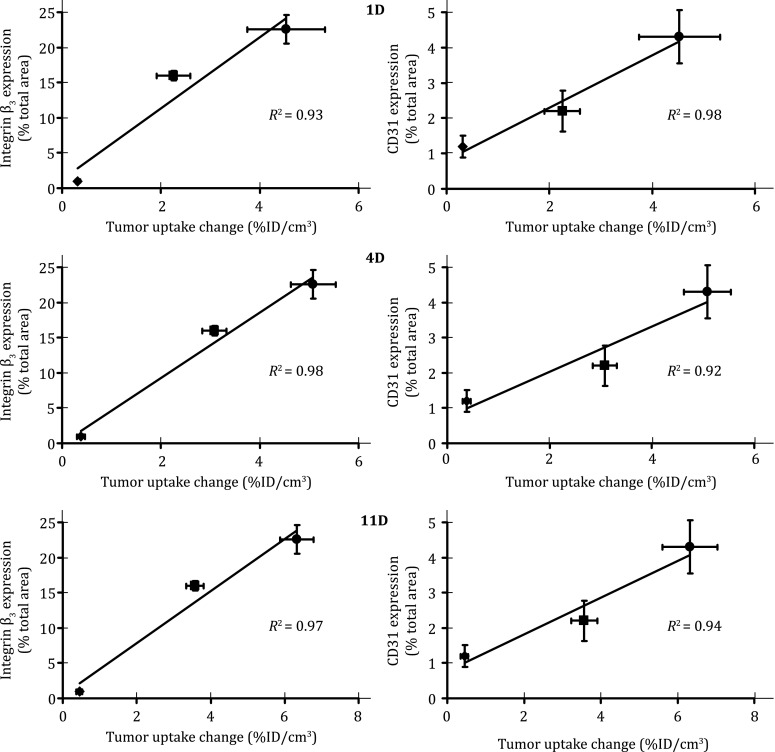
Linear relationship between the %ID/cm^3^ tumor uptake change at days 1 (*top*), 4 (*middle*) and 11 (*bottom*) after linifanib therapy and the expression levels of the α_v_β_3_ (*left*) and CD31 (*right*) in three tumor-bearing animal models. The %ID/cm^3^ tumor uptake values of ^99m^Tc-3P-RGD_2_ were calculated from SPECT/CT quantification and reported as the mean plus/minus standard error of the mean based on results from five animals (*n* = 5). The %ID/cm^3^ tumor uptake change was calculated by deducting the %ID/cm^3^ tumor uptake of ^99m^Tc-3P-RGD_2_ on days 1, 4, and 11 from its original value on −1 day (before linifanib therapy) in the same animal. The average %ID/cm^3^ tumor uptake change is used as the indicator of tumor response to linifanib treatment. The experimental data were adapted from Zheng et al. (2014)

^99m^Tc-3P-RGD_2_ has been used to monitor the tumor response to antiangiogenesis treatment with linifanib (ABT-869) (Ji et al. 2013b, d), a multi-targeted receptor tyrosine kinase inhibitor targeting vascular endothelial growth factor (VEGF) and platelet-derived growth factor (PDGF) receptors (Albert et al. 2006; Shankar et al. 2007; Wong et al. 2009; Zhou et al. 2009; Hernandez-Davies et al. 2011; Jiang et al. 2011; Tannir et al. 2011; Luo et al. 2012). We found that there was a significant decrease in tumor uptake (%ID/cm^3^) and *T*/*M* ratios of ^99m^Tc-3P-RGD_2_ in the xenografted U87MG model, while no significant changes in tumor uptake of ^99m^Tc-3P-RGD_2_ were seen in the PC-3 model after linifanib treatment (Ji et al. 2013d). The uptake changes in MDA-MB-435 tumors were between those observed in the U87MG and PC-3 models (Ji et al. 2013b). This is consistent with the tumor α_v_β_3_ expression levels (Zhou et al. 2011b). Highly vascularized tumors (e.g., U87MG) with higher level of α_v_β_3_ and CD31 have better tumor response to linifanib therapy than poorly vascularized tumors (e.g., PC-3) with low levels of α_v_β_3_ and CD31 (Fig. 10). Thus, ^99m^Tc-3P-RGD_2_ might be a screening tool to select appropriate patients who will benefits most antiangiogenic treatment. If the tumor has a high α_v_β_3_ expression, as indicated by high tumor uptake of ^99m^Tc-3P-RGD_2_ at the time of diagnosis, antiangiogenic therapy would more likely be effective. If the tumor has little α_v_β_3_ expression (low uptake of ^99m^Tc-3P-RGD_2_), antiangiogenic therapy would not be effective regardless the amount of antiangiogenic drug administered into the patient.

## MONITORING TUMOR METASTASIS

**Fig. 11:**
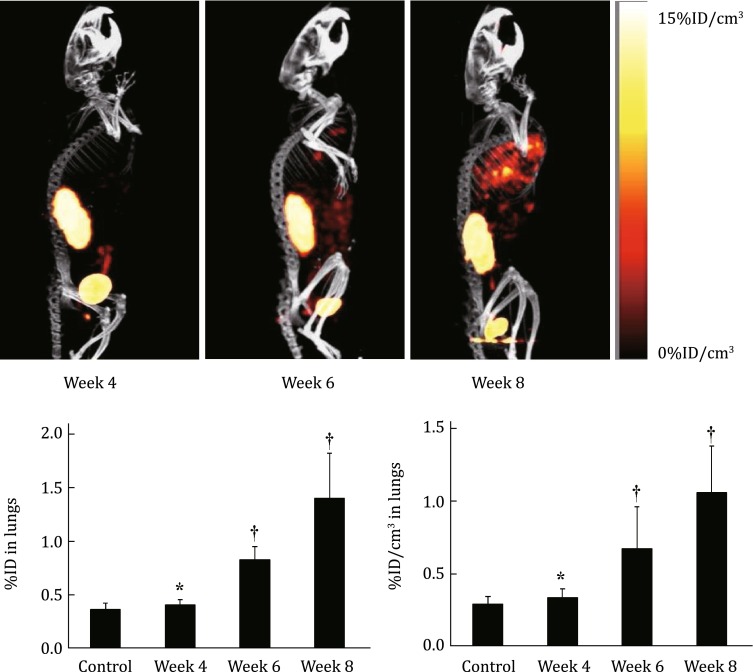
**Top**: The 3D views of SPECT/CT images of an athymic nude mouse at week 4, 6, and 8 after tail-vein injection of 1.0 × 10^6^ MDA-MB-231 cells. **Bottom**: The %ID (*left*) and %ID/cm^3^ (*right*) uptake values of ^99m^Tc-3P-RGD_2_ in the lungs obtained from SPECT/CT quantification in the athymic nude mice (*n* = 8) at week 4, 6, and 8 after tail-vein injection of 1.0 × 10^6^ MDA-MB-231 cells. Normal animals (*n* = 4) were used in the control group. ^†^ *p* < 0.05, significantly different from the control group; *p > 0.05, no significant difference from the control group. The imaging and SPECT quantification data were from Ji et al. (2013b)

^99m^Tc-3P-RGD_2_ SPECT/CT has been used as a noninvasive imaging tool to monitor the tumor growth and progression of breast cancer lung metastasis (Albert et al. 2006; Ji et al. 2013d). Figure 11 shows the SPECT/CT images of athymic nude mice (*n* = 8) with breast cancer lung metastasis. As expected, the SPECT/CT images showed no detectable metastatic breast tumor lesions in the lungs at week 4 (Fig. 11: top). By week 6, small breast cancer lesions started to appear in the mediastinum and lungs. At week 8, SPECT/CT images revealed many metastatic cancer lesions in both lungs (Albert et al. 2006). Figure 11 (bottom) compares the %ID (left) and %ID/cm^3^ (right) uptake values of ^99m^Tc-3P-RGD_2_ in the lungs. Even though the lung uptake of ^99m^Tc-3P-RGD_2_ (0.41 ± 0.05 %ID) at week 4 seemed to be higher than that in the control animals (0.36 ± 0.06 %ID), this difference was not significant (*p* > 0.05) within the experimental errors. At week 6, the tumor burden in the lungs became significant. The lung uptake of ^99m^Tc-3P-RGD_2_ was much higher (0.89 ± 0.12 %ID, *p* < 0.01) than that in the control group. By week 8, the uptake of ^99m^Tc-3P-RGD_2_ in the lungs was increase to 1.40 ± 0.42 %ID. In all cases, the lung size remained relatively unchanged (1.21–1.32 cm^3^) during the 8-week study period.

## CLINICAL EXPERIENCES WITH ^99m^Tc-3P-RGD_2_

The excellent in vivo tumor-targeting efficacy of ^99m^Tc-3P-RGD_2_ in animal models guaranteed its further clinical application. In a first-in-human study, ^99m^Tc-3P-RGD_2_ was investigated for its capability to noninvasively differentiate solitary pulmonary nodules (SPNs) (Ma et al. 2011). Among the 21 patients with SPNs, 15 (71%) were diagnosed as malignant while 6 (29%) were benign. The sensitivities for CT interpretation and ^99m^Tc-3P-RGD_2_ SPECT visual were 80% and 100%, respectively. All SPNs classified as indeterminate via CT can be sensitively diagnosed by ^99m^Tc-3P-RGD_2_ scintigraphy. ^99m^Tc-3P-RGD_2_ uptake in the malignant and benign nodules was well confirmed by ex vivo IHC staining of α_v_β_3_. These results demonstrated the feasibility of using ^99m^Tc-3P-RGD_2_ scintigraphy in differentiating SPNs (Ma et al. 2011). A multicenter study was performed in 70 patients with suspected lung lesions (Zhu et al. 2012). The results clearly demonstrated that ^99m^Tc-3P-RGD_2_ SPECT effectively detects lung malignancies, but with relatively low specificity. Whole-body planar scanning and chest SPECT are complementary for the evaluation of primary tumor and metastasis (Zhu et al. 2012). In a recently study, the potential of ^99m^Tc-3P-RGD_2_ SPECT in the detection of RAIR DTC lesions was conducted (Zhao et al. 2012). ^99m^Tc-3P-RGD_2_ SPECT identified all the target RAIR metastatic lesions, and there was a significant correlation between the mean tumor-to-background ratios and mean growth rates of target lesions. It is concluded that ^99m^Tc-3P-RGD_2_ imaging can be used for the localization and growth evaluation of RAIR lesions, thus providing a promising imaging strategy to monitor the efficacy of antiangiogenic therapy (Zhao et al. 2012). ^99m^Tc-3P-RGD_2_ SPECT was also evaluated and compared to ^99m^Tc-MIBI for the capability to assess the breast cancer lessons (Ma et al. 2014). It was found that the mean T/NT ratio of ^99m^Tc-3P-RGD_2_ in malignant lesions was significantly higher than that in benign lesions (3.54 ± 1.51 vs. 1.83 ± 0.98, *p* < 0.001). The sensitivity, specificity, and accuracy of ^99m^Tc-3P-RGD_2_ SMM were 89.3%, 90.9%, and 89.7%, respectively, with a T/NT cut-off value of 2.40. The mean T/NT ratio of ^99m^Tc-MIBI in malignant lesions was also significantly higher than that in benign lesions (2.86 ± 0.99 vs. 1.51 ± 0.61, *p* < 0.001). The sensitivity, specificity, and accuracy of ^99m^Tc-MIBI SMM were 87.5%, 72.7%, and 82.1%, respectively, with a T/NT cut-off value of 1.45. According to the ROC analysis, the area under the curve for ^99m^Tc-3P-RGD_2_ SMM (area = 0.851) was higher than that for ^99m^Tc-MIBI SMM (area = 0.781), but the statistical difference was not significant.

## CLINICAL EXPERIENCES WITH ^18^F-ALFATIDE AND ^18^F-ALFATIDE II

^18^F-labeled RGD compounds suffer from multistep and time-consuming synthetic procedures, which will limit their clinic availability. To overcome this shortcoming, the Al(NOTA) chelate has been used for ^18^F-labeling of P-RGD_2_ (Lang et al. 2011). The application of NOTA-AlF chelation chemistry and kit formulation allows one-step ^18^F-labeling. Under the optimal conditions, the radiotracer [^18^F]AlF(NOTA-P-RGD_2_) (denoted as ^18^F-Alfatide) was prepared in relatively high yield (42.1 ± 0.02) with more than 95% radiochemical purity. The whole radiosynthesis including post-labeling chromatographic purification was accomplished within 20 min. Nine patients with a primary diagnosis of lung cancer were examined by both static and dynamic PET imaging with ^18^F-alfatide, and one tuberculosis patient was investigated using both ^18^F-alfatide and ^18^F-FDG imaging. It was found that ^18^F-alfatide PET identified all tumors, with mean standardized uptake values of 2.90 ± 0.10. Tumor-to-muscle and tumor-to-blood ratios were 5.87 ± 2.02 and 2.71 ± 0.92, respectively. It was concluded that PET scanning with ^18^F-alfatide allows specific imaging of avb3 expression with good contrast in lung cancer patients.

## CONCLUSIONS

Over the last several years, many multimeric cyclic RGD peptides have been used to increase the radiotracer tumor-targeting capability. The fact that radiolabeled (^18^F, ^99m^Tc, ^111^In, ^64^Cu, and ^68^Ga) cyclic RGD peptides to target multiple integrins (α_v_β_3_, α_v_β_5_, α_5_β_1_, α_6_β_4_, α_4_β_1_, and α_v_β_6_) will help to improve their tumor uptake due to the “increased receptor population.” In order to achieve bivalency, the distance between two cyclic RGD motifs must be long enough so that they will be able to bind the two adjacent α_v_β_3_ sites simultaneously. Multimerization increases the uptake of radiolabeled multimeric cyclic RGD peptides in both the tumor and normal organs, and also their tumor retention times. Among the radiotracers evaluated in tumor-bearing models, the radiolabeled cyclic RGD dimers (e.g., 2P-RGD_2_, 3P-RGD_2,_ 2G-RGD_2_, 3G-RGD_2_, and Galacto-RGD_2_) show the most promising results with respect to their tumor uptake and *T*/*B* ratios. ^99m^Tc-3P-RGD_2_, ^18^F-Alfatide, and ^18^F-Alfatide II are currently under clinical investigation for tumor imaging by SPECT or PET. ^99m^Tc-3P-RGD_2_ offers significant advantages over both ^18^F-Alfatide and ^18^F-Alfatide II because it could be routinely prepared in high yield and radiochemical purity (>95%) without post-labeling chromatographic purification and clinical availability of ^99^Mo-^99m^Tc generators. However, SPECT has limitations in quantification of radiotracer uptake, the speed of dynamic imaging, spatial resolution, and tissue attenuation.

## General terms

DCE-MRI: Dynamic contrast-enhanced magnetic resonance imaging
FITC: Fluorescein isothiocyanate isomer I
^18^F-FDG: 2-Deoxy-2-(^18^F)fluoro-d-glucose
IHC: Immunohistochemistry
MRI: Magnetic resonance imaging
PET: Positron emission tomography
PDGFR: Platelet-derived growth factor receptors
SPECT: Single-photon emission computed tomography
VEGFR: Vascular endothelial growth factor receptors

## Chelators

DOTA: 1,4,7,10-Tetraazacyclododecane-1,4,7,10-tetracetic acid
HYNIC: 6-Hydazinonicotinic acid
NOTA: 1,4,7-triazacyclononane-1,4,7-triacetic acid

## Cyclic peptides

Galacto-RGD_2_: Glu[cyclo[Arg-Gly-Asp-d-Phe-Lys(SAA-PEG_2_-(1,2,3-triazole)-1-yl-4-methylamide)]]_2_ (SAA = 7-amino-l-glycero-l-galacto-2,6-anhydro-7-deoxyheptanamide, and PEG_2_ = 3,6-dioxaoctanoic acid)
P-RGD: PEG_4_-c(RGKfD) = cyclo(Arg-Gly-Asp-d-Phe-Lys(PEG_4_)) (PEG_4_ = 15-amino-4,7,10,13-tetraoxapentadecanoic acid)
RGD_2_: E[c(RGDfK)]_2_ = Glu[cyclo(Arg-Gly-Asp-d-Phe-Lys)]_2_
P-RGD_2_: PEG_4_-E[c(RGDfK)]_2_ = PEG_4_-Glu[cyclo(Arg-Gly-Asp-d-Phe-Lys)]_2_
2G-RGD_2_: E[G_3_-c(RGDfK)]_2_ = Glu[cyclo[Arg-Gly-Asp-d-Phe-Lys(G_3_)]]_2_ (G_3_ = Gly-Gly-Cly)
2P-RGD_2_: E[PEG_4_-c(RGDfK)]_2_ = Glu[cyclo[Arg-Gly-Asp-d-Phe-Lys(PEG_4_)]]_2_
3G-RGD_2_: G_3_-E[G_3_-c(RGDfK)]_2_ = G_3_-Glu[cyclo[Arg-Gly-Asp-d-Phe-Lys(G_3_)]]_2_
3P-RGD_2_: PEG_4_-E[PEG_4_-c(RGDfK)]_2_ = PEG_4_-Glu[cyclo[Arg-Gly-Asp-d-Phe-Lys(PEG_4_)]]_2_
3P-RGK_2_: PEG_4_-E[PEG_4_-c(RGDfK)]_2_ = PEG_4_-Glu[cyclo[Arg-Gly-Lys(PEG_4_)-d-Phe-Asp]]_2_)
RGD_4_: E{E[c(RGDfK)]_2_}_2_ = Glu{Glu[cyclo(Arg-Gly-Asp-d-Phe-Lys)]_2_}_2_
6G-RGD_4_: E{G_3_-E[G_3_-c(RGDfK)]_2_}_2_ = Glu{G_3_-Glu[cyclo(Lys(G_3_)-Arg-Gly-Asp-d-Phe)]-cyclo(Lys(G_3_)-Arg-Gly-Asp-d-Phe)}-{PEG_4_-Glu[cyclo(Lys(G_3_)-Arg-Gly-Asp-d-Phe)]-cyclo(Lys(G_3_)-Arg-Gly-Asp-d-Phe)}
6P-RGD_4_: E{PEG_4_-E[PEG_4_-c(RGDfK)]_2_}_2_ = Glu{PEG_4_-Glu[cyclo(Lys(PEG_4_)-Arg-Gly-Asp-d-Phe)]-cyclo(Lys(PEG_4_)-Arg-Gly-Asp-d-Phe)}-{PEG_4_-Glu[cyclo(Lys(PEG_4_)-Arg-Gly-Asp-d-Phe)]-cyclo(Lys(PEG_4_)-Arg-Gly-Asp-d-Phe)}

## Bioconjugates of cyclic peptides

DOTA-RGD: DOTA-c(RGDfK)
DOTA-P-RGD: DOTA-PEG_4_-c(RGDfK)
DOTA-RGD_2_: DOTA-E[c(RGDfK)]_2_
DOTA-P-RGD_2_: DOTA-PEG_4_-E[c(RGDfK)]_2_
DOTA-2G-RGD_2_: DOTA-E[G_3_-c(RGDfK)]_2_
DOTA-2P-RGD_2_: DOTA-E[PEG_4_-c(RGDfK)]_2_
DOTA-3G-RGD_2_: DOTA-G_3_-E[G_3_-c(RGDfK)]_2_
DOTA-3P-RGD_2_: DOTA-PEG_4_-E[PEG_4_-c(RGDfK)]_2_
DOTA-3P-RGK_2_: DOTA-PEG_4_-E[PEG_4_-c(RGDfK)]_2_
DOTA-Galacto-RGD_2_: DOTA-Glu[cyclo[Arg-Gly-Asp-d-Phe-Lys(SAA-PEG_2_-(1,2,3-triazole)-1-yl-4-methylamide)]]_2_
DOTA-RGD_4_: DOTA-E{E[c(RGDfK)]_2_}_2_
DOTA-6G-RGD_4_: DOTA-E{G_3_-E[G_3_-c(RGDfK)]_2_}_2_
DOTA-6G-RGD_4_: E{PEG_4_-E[PEG_4_-c(RGDfK)]_2_}_2_
FITC-3P-RGD_2_: FITC-PEG_4_-E[PEG_4_-c(RGDfK)]_2_
FITC-3P-RGK_2_: FITC-PEG_4_-E[PEG_4_-c(RGDfK)]_2_
HYNIC-RGD_2_: HYNIC-E[c(RGDfK)]_2_
HYNIC-P-RGD_2_: HYNIC-PEG_4_-E[c(RGDfK)]_2_
HYNIC-2G-RGD_2_: HYNIC-E[G_3_-c(RGDfK)]_2_
HYNIC-2P-RGD_2_: HYNIC-E[PEG_4_-c(RGDfK)]_2_
HYNIC-3G-RGD_2_: HYNIC-G_3_-E[G_3_-c(RGDfK)]_2_
HYNIC-3P-RGD_2_: HYNIC-PEG_4_-E[PEG_4_-c(RGDfK)]_2_
HYNIC-Galacto-RGD_2_: HYNIC-Glu[cyclo[Arg-Gly-Asp-d-Phe-Lys(SAA-PEG_2_-(1,2,3-triazole)-1-yl-4-methylamide)]]_2_
HYNIC-RGD_4_: HYNIC-E{E[c(RGDfK)]_2_}_2_
NOTA-P-RGD_2_: NOTA-PEG_4_-E[c(RGDfK)]_2_
NOTA-2G-RGD_2_: NOTA-E[G_3_-c(RGDfK)]_2_
NOTA-2P-RGD_2_: NOTA-E[PEG_4_-c(RGDfK)]_2_
NOTA-3G-RGD_2_: NOTA-G_3_-E[G_3_-c(RGDfK)]_2_
NOTA-3P-RGD_2_: NOTA-PEG_4_-E[PEG_4_-c(RGDfK)]_2_

## Radiolabeled cyclic RGD peptides

^18^F-Alfatide: [^18^F]AlF(NOTA-P-RGD_2_)
^18^F-Alfatide II: [^18^F]AlF(NOTA-2P-RGD_2_)
^18^F-Galacto-RGD: 2-[^18^F]fluoropropanamide c(RGDfK(SAA), SAA = 7-amino-L-glyero-L-galacto-2,6-anhydro-7-deoxyheptanamide)
^64^Cu-P-RGD_2_: ^64^Cu(DOTA-P-RGD_2_)
^64^Cu-2G-RGD_2_: ^64^Cu(DOTA-2G-RGD_2_)
^64^Cu-2P-RGD_2_: ^64^Cu(DOTA-2P-RGD_2_)
^64^Cu-3G-RGD_2_: ^64^Cu(DOTA-3G-RGD_2_)
^64^Cu-3P-RGD_2_: ^64^Cu(DOTA-3P-RGD_2_)
^68^Ga-3G-RGD_2_: ^68^Ga(DOTA-3G-RGD_2_)
^68^Ga-3P-RGD_2_: ^68^Ga(DOTA-3P-RGD_2_)
^111^In-P-RGD: ^111^In(DOTA-P-RGD)
^111^In-P-RGD_2_: ^111^In(DOTA-P-RGD_2_)
^111^In-2G-RGD_2_: ^111^In(DOTA-2G-RGD_2_)
^111^In-2P-RGD_2_: ^111^In(DOTA-2P-RGD_2_)
^111^In-3G-RGD_2_: ^111^In(DOTA-3G-RGD_2_)
^111^In-3P-RGD_2_: ^111^In(DOTA-3P-RGD_2_)
^111^In-Galacto-RGD_2_: ^111^In(DOTA-Galacto-RGD_2_)
^111^In-6G-RGD_4_: ^111^In(DOTA-6G-RGD_4_)
^111^In-6P-RGD_4_: ^111^In(DOTA-6P-RGD_4_)
^99m^Tc-Galacto-RGD_2_: [^99m^Tc(HYNIC-Galacto-RGD_2_)(tricine)(TPPTS)])
^99m^Tc-RGD_2_: [^99m^Tc(HYNIC-RGD_2_)(tricine)(TPPTS)])
^99m^Tc-P-RGD_2_: [^99m^Tc(HYNIC-P-RGD_2_)(tricine)(TPPTS)]
^99m^Tc-2G-RGD_2_: [^99m^Tc(HYNIC-2G-RGD_2_)(tricine)(TPPTS)]
^99m^Tc-2P-RGD_2_: [^99m^Tc(HYNIC-2P-RGD_2_)(tricine)(TPPTS)]
^99m^Tc-3G-RGD_2_: [^99m^Tc(HYNIC-3G-RGD_2_)(tricine)(TPPTS)]
^99m^Tc-3P-RGD_2_: [^99m^Tc(HYNIC-3P-RGD_2_)(tricine)(TPPTS)]
^99m^Tc-RGD_4_: [^99m^Tc(HYNIC-RGD_4_)(tricine)(TPPTS)])
